# Review of Dietary Recommendations for Twin Pregnancy: Does Nutrition Science Keep Up with the Growing Incidence of Multiple Gestations?

**DOI:** 10.3390/nu14061143

**Published:** 2022-03-08

**Authors:** Regina Ewa Wierzejska

**Affiliations:** Department of Nutrition and Nutritional Value of Food, National Institute of Public Health NIH—National Research Institute, Chocimska St. 24, 00-791 Warsaw, Poland; rwierzejska@pzh.gov.pl

**Keywords:** twin pregnancy, nutrition, requirements, supplementation, pregnancy outcomes

## Abstract

Recommendations for nutrition and the use of dietary supplements for pregnant women are updated on regular basis but it remains to be seen to what extent they may be applicable in twin pregnancies. The aim of this narrative review is to present the current state of knowledge about the energy and nutrient demand in twin pregnancy. There is general consensus in literature that the energy demand is higher than in a singleton pregnancy, but there is a lack of position statements from scientific societies on specific energy intake that is required. In turn, recommended maternal weight gain, which favors the normal weight of the neonate, has been determined. There is even a larger knowledge gap when it comes to vitamins and minerals, the body stores of which are theoretically used up faster. The greatest number of studies so far focused on vitamin D, and most of them concluded that its concentration in maternal blood is lower in twin as compared to singleton pregnancy. Few randomized studies focus on iron supplementation and there are no other studies that would assess dietary interventions. In light of a growing incidence of multiple pregnancies, more studies are necessary to establish the nutritional demands of the mother and the course of action for adequate supplementation.

## 1. Introduction

Over the past 30 years, there has been a considerable increase in the number of multiple pregnancies worldwide, which mainly applies to dizygotic pregnancies. In the USA, in 2009 compared to the 1980s, the number of twin pregnancies increased by 76%; the situation was similar in Australia (66%) [1,2]. Currently, it is estimated that in the United States, twin births account for 3.0–3.5% of all births (one twin birth out of approx. 30 births), much like in France and Australia—more than 3%. A lower proportion is observed in Central Africa (1.8%) and Great Britain (1.5%), while in East Asia and Oceania, the incidence of twin pregnancies has been stable over the years and amounts to <1% of all births [2,3,4,5,6,7,8,9]. This upward trend has also been observed in Poland. According to the Yearbook of Statistics Poland, in 1990, twin pregnancies accounted for 1% of all births, while in 2018, 1.3%, which corresponds to 4925 births [10,11].

Among the reasons for the increased number of multiple pregnancies, several factors are taken into consideration. The main one is the use of assisted reproductive technology, which is probably responsible for two-thirds of said increase; the other is the more advanced age of women giving birth, which accounts for approximately one-quarter of the increase [9,12,13,14,15,16]. As an example, in 2002 in the USA, the number of twin pregnancies in women aged ≥ 40 was 11 times higher compared to 1980 [17]. In Poland, in 1990, twin pregnancies after the age of 35 constituted 11.5% of all twin pregnancies, while in 2018, it was 23.3% [10,11]. The mother’s pre-pregnancy obesity can also be of considerable importance. It is estimated that with a *body mass index* (BMI) of >30, the risk of a twin pregnancy grows by 40%, which, in times of globally increasing obesity, can be an important factor conducive to multiple pregnancies [9,14,15,16]. Literature also investigates pre-pregnancy folic acid use and mandatory folic acid food fortification introduced in many countries [2,18,19,20]. 

A multiple pregnancy is associated with an increased risk of its abnormal course. This mostly applies to pre-eclampsia, hypertension, diabetes, iron-deficiency anemia and pre-term birth that affects 50–60% of twin pregnancies. For newborns, the risk mainly concerns low birth weight (<2500 g) and the resulting perinatal death [6,13,21,22,23,24]. When it comes to twin pregnancies, low birth weight applies to approx. 50% newborns, a birth weight of <1500 g applies to 10%, while for newborns from a singleton pregnancy, it is 6% and 1%, respectively [15]. 

Although nutritional recommendations for pregnant women are quite detailed and do not specify the type of pregnancy [25,26,27], these refer to women who expect one baby by default. For multiple pregnancies, literature shows a clear gap in knowledge and, thereby, a lack of separate guidelines. Actually, multiple pregnancies are rarely the subject matter of scientific studies. An estimation of the number of randomized studies carried out in the years 2012–2016 with regard to pregnancy complications demonstrates that, in reference to fetal growth restriction, gestational diabetes and pre-eclampsia, there is not one study concerned with multiple pregnancies only. A slightly better situation is seen for studies associated with pre-term birth, where out of all the studies, 18% applied to multiple pregnancies, while 17% to multiple and singleton pregnancies together [13]. 

The aim of this narrative review is to present the current state of knowledge on the energy requirement, optimal gestational weight gain, vitamin, mineral and other nutrient requirements of women pregnant with twins. 

## 2. Material and Methods

Articles written in English were searched in PubMed and Medline until September 2021 using keywords: “twin pregnancy”, “twin gestation”, “multiple pregnancy”, accompanied by the terms such as: “vitamins”, “minerals”, “nutrients”, “nutrition”, “diet”, “gestational weight gain”. Articles such as randomized controlled trials, other original studies, meta-analyses of studies, position statements by scientific societies, guidelines from the Food and Agriculture Organization, World Health Organization and United Nations University, as well as review papers were taken into account. 

## 3. Results

### 3.1. Energy Requirement of Women Pregnant with Twins

Energy requirement in pregnancy is defined as a dietary energy value needed to provide the optimum development of maternal tissue and fetal growth [28]. Pregnancy increases resting energy expenditure due to the development of the placenta and the fetus as well as the enhanced maternal heart and lung function [29]. Basal metabolic rate in women with a singleton pregnancy increases by approx. 5% in the first trimester of pregnancy, by 11% in the second trimester and 24% in the third trimester [30,31]. When it comes to a twin pregnancy, scientific data suggest that, in the third trimester, basal metabolic rate increases by additional 10%. Apart from energy expenditure by the mother’s body, the total energy requirement also includes the accumulation of energy in the form of fatty tissue, referred to as energy storage [32].

The diet’s energy value determines the gestational body weight gain [15,33]. According to expert guidelines from the Food and Agriculture Organization, World Health Organization and United Nations University (FAO/WHO/UNU), standards for singleton pregnancies assume that the normal body weight gain is on average 12 kg. In such a case, the overall energy cost of pregnancy is estimated at 77,000 kcal (on average 275 kcal per day) and this additional energy should be properly distributed over the individual trimesters of pregnancy [30]. The worldwide recommendations for energy intake in singleton pregnancies are quite similar. According to guidelines from North America, Europe, Australia and New Zealand, the higher energy requirement starts from the second trimester (by 335–360 kcal/day) to reach 450–475 kcal in the third trimester. Only Japan recommends a small increase of 50 kcal already in the first trimester, while in the subsequent trimesters, it is 250 and 500 kcal/day higher, respectively [34]. 

In terms of a twin pregnancy, there is general consensus in literature that, due to the higher weight of maternal tissues, the development of two fetuses and increased energy expenditure, the energy requirement is even larger [14,15,33]. It needs to be noted, however, that due to a lack of studies, these are only theoretical assumptions, and the actual energy needs of women pregnant with twins remain unknown [14,17]. Taking the target body weight gain of 20 kg in women with a twin pregnancy, some estimate that throughout the entire pregnancy, additional 35,000 kcal need to be supplied in excess of what is already needed for a singleton pregnancy. Considering the higher restrictions in physical activity, this means that 150 kcal need to be additionally consumed per day compared to the recommendations for singleton pregnancies [14,15]. Gandhi et al., in their study among women with a dichorionic diamniotic twin pregnancy, estimated that up until 30–32 weeks of gestation, weight gain comprised about 6 kg of fat mass and 2 kg of protein, equal to a mean energy deposition of 67,042 kcal. The third trimester energy requirement estimated by the authors increased by 29% compared to the first trimester—from 2257 ± 325 kcal/d in the first trimester to 2906 ± 350 kcal/d in the third trimester [33]. The authors argue that, in order to cover the increased energy expenditure and ensure the normal maternal body weight gain, the energy intake in women pregnant with twins should grow by an average of 700 kcal daily in the second and third trimester compared to the first trimester. On the other hand, the results of the Canadian *Higgins Nutrition Intervention Program* indicate that, after the 20th week of pregnancy, it is advisable to consume additional 1000 calories per day and 50 g of protein (by convention, 500 kcal and 25 g of protein per each child) compared to women who are not pregnant. Such a diet resulted in the increased body weight of the twins by an average of 80 g and the reduced number of pre-term births by 30%, compared to the twins of mothers from the control group [35]. 

Analogically, as in a singleton pregnancy, the diet’s energy value should be adapted to the mother’s pre-pregnancy BMI. Luke reports that the energy requirement in preconceptionally underweight women pregnant with twins amounts to 4000 kcal, in women with normal pre-pregnancy weight—3500 kcal, in overweight women—3250 kcal, while in obese women—3000 kcal [17]. Similar values are given by Whitaker et al., who claim that women with normal pre-pregnancy BMI need 3000–3500 kcal, overweight women—3250 kcal and obese women—2700–3000 kcal [7], while in the light of the guidelines from the Alberta Health Services, the diet’s energy value in a twin pregnancy should amount to 3000–4000 kcal, with the lowest value applying to women with an excessive preconceptional BMI and the highest to slim women [36]. A different group of experts claims that women pregnant with twins with a normal pre-pregnancy body weight need 40–45 kcal/kg bw/day in the first trimester, with a possible modification in the next trimesters, in order to ensure the normal maternal body weight gain [37]. Based on this premise, assuming the body weight of a mother-to-be is, for example, 70 kg, this would mean that her diet in the first trimester should amount to 2800–3150 kcal per day. 

Just like in singleton pregnancies, determining the optimum energy value of the diet presents a particular challenge in obese women who are at risk of an excessive body weight gain. At the moment, there is a lack of studies among women pregnant with twins, but, based on studies among women with a singleton pregnancy, it can be deliberated whether or not a small energy deficit in the diet might be an effective solution preventing an excessive body weight gain. One of such studies demonstrated that the normal body weight gain in women with pre-pregnancy obesity was achieved when the diet’s energy value was on average 125 kcal/day lower than the body’s energy expenditure, while the consumption of 186 kcal more compared to the energy expenditure led to an excessive body weight gain. The authors of said study therefore recommend that the energy value of the diet of pregnant women with obesity should not exceed the body’s total energy expenditure [38]. An estimation of pregnant women’s energy requirements carried out by Most et al., show that in order to achieve the normal body weight gain, obese women not only should stop accumulating energy in the form of fatty tissue, but should even derive approximately 160 kcal from their body’s energy stores per day. In practice, it means supplying 5% less energy with the diet compared to the energy expenditure [28]. Given that energy accumulated during pregnancy in the form fatty tissue is to serve as an energy reserve for the postpartum period and lactation and that in countries not affected by the problem of starvation and access to food, it seems that overweight women pregnant with twins should not store energy in the form of fatty tissue because energy stores in the body are sufficient for the pregnancy and subsequent lactation. 

At this point, it is worth citing a very interesting study concerned with dietary advice for American women pregnant with twins. It demonstrated that only 30% women received recommendations regarding the diet’s calorie value. Among them, 6.4% stated that they were told to consume 300–500 kcal/day more, while 4.6% women said that an additional 600–1500 kcal were recommended. Further 8.1% women were advised that the total energy value of their diet should amount to 2500–3500 kcal per day; other recommended values were 2000–2500 kcal (4.6% women) and 1600–1800 kcal (2.3% women) [7]. This shows very diverse approaches adopted by dieticians and the complexity of this problem.

It needs to be acknowledged that women pregnant with twins suffer from nausea, vomiting and early satiety much more often than women with a singleton pregnancy, which can cause difficulties in adhering to the higher requirements [36,39,40]. A study by Morley et al., demonstrates that the median of daily energy intake by women pregnant with twins at 29–35 weeks of gestation ranged from 2363 to 2388 kcal [41]. Theoretically, it would indicate an energy deficit in the diet, which actually should not come as a surprise because energy intake considerably lower than the guidelines is commonly observed in singleton pregnancies as well. In the light of a 2012 meta-analysis of observational studies, average energy intake in pregnancy in the USA and Canada amounted to 2199 kcal, in Europe—2194 kcal, while in Japan only 1839 kcal [34]. Similar results were obtained in a meta-analysis of 2016, where average energy intake was 1940 kcal/day in the first trimester and 2052 kcal/day in the third trimester [42].

### 3.2. Body Weight Gain

It is generally believed that the energy value of pregnant women’s diet is a marker of body weight gain, and it is also an important factor determining the course of pregnancy. [43,44,45]. For example, too low a body weight gain increases the risk of pre-term birth, while an excessive body weight gain—the risk of gestational diabetes and hypertension [46,47]. Both in a singleton and twin pregnancy, the maternal body weight gain also correlates with the newborns’ birth weight. Mothers with a low body weight gain more frequently give birth to small-for-gestational-age infants, while among the newborns of mothers who gained too much weight, macrosomia is more common [14,46,48,49]. For twin pregnancies, it is suggested that the body weight gain in the individual trimesters of pregnancy can be more important than the total weight gain [50]. According to some experts, the body weight gain of mothers with a twin pregnancy starts sooner than those with a singleton pregnancy [15], while other experts claim that until 18 weeks, the body weight gain of women is the same and it is only after this period that it significantly increases in the case of a twin pregnancy [21]. The mother’s body weight gain at the very beginning of gestation can favorably influence the development and functioning of the placenta. It is important because, in multiple pregnancies, the placenta probably matures and ages faster, which reduces the period of optimum nutrient supply to the fetus [15,36].

Although many experts argue that the maternal body weight gain in the first half of a twin pregnancy is particularly important in terms of the newborns’ birth weight [36,40], study results in that regard are inconclusive. According to Brown et al., it is best when not only a pregnant woman does not lose weight in the first trimester, but puts on approximately 1.8–2.7 kg [14]. Roem reports that, if the body weight gain is less than 6 kg until 24 weeks of gestation, then the risk of impaired fetal growth rises [40], while Luke et al., suggest that, in order to avoid giving birth to low-birth-weight infants, the body weight gain until 24 weeks of pregnancy needs to be approximately 11 kg [51]. It is assumed that, until the 20th week of pregnancy, each 1 kg of mother’s body weight gain/week increases the weight of both newborns by 65 g. Such a body weight gain between 20- and 28-weeks results in the child’s body weight increase of 37 g, while after 28 weeks of pregnancy—only 16 g [52]. A different set of data, however, were provided by Hinkle et al., who found that only the relationship between the newborns’ birth weight and the mother’s body weight gain in the second trimester was statistically significant. Each kilogram put on by the mother between 14 and 20 weeks of gestation meant that the body weight of each newborn increased by an average of 133 g, while each kilogram of body weight gain between 21 and 27 weeks increased the average body weight of the newborns by 57 g [50]. In a study by Pettit et al., a low body weight gain between the 20th and the 28th week of pregnancy turned out to be the strongest risk factor for a birth before the 32nd week [23].

When it comes to the total body weight gain of pregnant women, many countries, including Poland, adopted the guidelines of the American Institute of Medicine of 2009, which were developed to minimize the risk of giving birth to an infant that is small or large for gestational age [53,54]. According to the guidelines, the body weight gain of women pregnant with twins depends on their pre-pregnancy BMI, just like for a singleton pregnancy. Based on the course of pregnancy in women who gave birth after the end of the 36th week of pregnancy to twins weighing ≥ 2500 g, it was determined that it is optimal for women with a normal body weight to put on 17 to 25 kg, overweight women—14–23 kg, while obese women—11–19 kg [21,46,53]. It needs to be emphasized that such a body weight gain in women pregnant with twins who had a normal pre-pregnancy body weight is 47–56% higher compared to women expecting one child, while in obese women—even 111–120% higher. Additionally, according to the body weight gain chart developed in 2018 by Hutcheon et al., based on a group of 1109 American women pregnant with twins who gave birth after the end of the 36th week of pregnancy, the body weight gain should drop as the preconceptional BMI increases. At 20 weeks of pregnancy, in women with a normal pre-pregnancy body weight, the body weight gain at the 50th percentile amounted to 6.8 kg, while in overweight women—5.7 kg and in obese women—3.6 kg. The same trend was observed at 37 weeks of pregnancy when the body weight gain in the above groups of women was 19.8 kg, 18.1 kg and 14.4 kg, respectively [21]. On the other hand, according to Luke’s guidelines, in order for twins to reach their birth weight at 36–38 weeks of pregnancy, which is 2700–2800 g, the optimum body weight gain for a preconceptionally underweight mother is 22.7–28.1 kg, a mother with normal body weight—18.1–24.5 kg, an overweight mother—17.2–21.3 kg, while for a preconceptionally obese mother—13.2–17.2 kg [17]. Other experts report that, until the 37th week of pregnancy, the body weight gain that reduces the risk of the newborns’ low birth weight is 16–20.5 kg, with the higher value applying to preconceptionally underweight women and the lower value to overweight women [14,15,40]. Although women with a singleton pregnancy with pre-pregnancy Class 2 obesity (BMI ≥ 35) are advised that their total body weight gain be less than 5 kg, there are no guidelines yet for women pregnant with twins [28].

In literature, the body weight gain in women with a twin pregnancy was mainly assessed in the context of the American guidelines. In a study by Bodnar et al., carried out in a group of more than 27,000 women pregnant with twins, a body weight gain that was considerably lower than the recommended one increased the risk of small-for-gestational-age infants, while a body weight gain much in excess of the recommended one increased the risk of large-for-gestational-age infants. Furthermore, it was found that the body weight gain of women with pre-pregnancy underweight or normal body weight had a U-shaped relationship with the risk of giving birth before the end of the 32nd week of pregnancy [22]. The results of a study by Lal and Kominiarek carried out in a group of more than 2600 women pregnant with twins who were underweight or had normal weight before pregnancy and who did not reach the minimum body weight gain demonstrated that these women more frequently gave birth to children weighing less than 2500 g and less than 1500 g compared to women with a body weight gain in excess of the recommended one (57.2% and 10.6% vs. 36.2% and 4.3%). The risk of low birth weight in both infants grew as the body weight gain of the mother decreased in every group of women, while an excessive body weight gain turned out to be a protective factor in that regard [6]. Similarly, a study by Fox et al., demonstrated that a body weight gain in excess of the American guidelines increased the infants’ birth weight but it did not increase the risk of gestational diabetes, hypertension and pre-eclampsia [55]. However, according to Lal and Kominiarek, in women with an excessive body weight gain, there was a higher incidence of pre-eclampsia and pregnancy-induced hypertension [6], while Ozcan et al., found that an excessive body weight gain in women pregnant with twins increased the risk of hypertension and macrosomia [56]. Based on their literature review, Pecheux et al., claim that the American guidelines for the body weight gain in women pregnant with twins should be used in daily obstetrical practice because such a body weight gain reduces the risk of pre-term birth, low birth weight and pregnancy-induced hypertension [47]. Asian countries might be an exception due to the lower height of women. As Obata et al., indicate, the body weight gain in Japanese women pregnant with twins that led to a normal birth weight of both infants was considerably lower than the American guidelines in more than 70% women and amounted to 11.5–16.5 kg in preconceptionally underweight women, 10.3–16.0 kg in women with a normal body weight, 6.9–14.7 kg in overweight women and 2.2–11.7 kg in obese women [46].

Some argue that the body weight gain guidelines for pregnant women are only theoretical. United Kingdom belongs to the group of countries which question the validity of routine weigh-ins for pregnant women and maternal weight policies. The United Kingdom’s National Institute for Health and Clinical Excellence firmly claims that data on the link between maternal weight gain and neonatal health are inconclusive and that the limits give rise to unnecessary concern among the mothers-to-be [57,58]. It is difficult to oppose such an approach firmly because, in practice, few women actually meet the requirements set for them. When it comes to singleton pregnancies, a meta-analysis of studies indicates that 51% women in Europe and the USA and 37% women in Asia exceed the American body weight gain guidelines, while 21%, 18% and 31% women in these world regions, respectively, do not reach the minimum body weight gain [48]. Similar conclusions apply to twin pregnancies, although there are definitely fewer studies. In a study by Gandhi et al., 40% of American women with a normal pre-pregnancy body weight met the guidelines of the Institute of Medicine, 20% gained too little weight, while 40% gained too much weight; among overweight women, however, only 20% women met the guidelines and the remaining 80% gained too much weight [33]. In Canada, 43% of women pregnant with twins met the American guidelines, 27% gained too little and 30% gained too much weight [59].

### 3.3. Vitamin Requirement

Many experts claim that the requirement for vitamins and minerals is higher in a twin pregnancy than it is in a singleton pregnancy, but it is not currently established and this view is based on a hypothesis that the maternal body reserves are used up faster [14,15,17,37,39,60]. For most vitamins and minerals, there either no studies that would assess their concentration in the body of a woman pregnant with twins or there are very few such studies (Table 1 summarizes the studies included in this paper). First of all, there are no randomized studies relating to the effects of dietary interventions on the course of pregnancy, which means there are no grounds for reliable guidelines as regards vitamin and mineral intake and dietary supplementation [4,7,13,61]. The only dietary supplementation guidelines for women pregnant with twins known to the author were published in 1990 by the American Institute of Medicine and Alberta Health Services in Canada (2018) [14,36].

#### 3.3.1. Folic Acid

Folic acid is an essential nutrient in pregnancy, which is more required, for example, due to the increased cell division process [2]. According to the American Institute of Medicine and European Food Safety Authority, the requirement for pregnant women is 600 µg/day [71]. Due to problems with covering this requirement by diet alone, global guidelines indicate it is necessary to supplement folic acid at a minimum of 400 µg per day [20]. It is estimated that the risk of folate-deficiency anemia in a twin pregnancy is eight times higher than in a singleton pregnancy [36], which raises the question of whether the requirements for a woman with a twin pregnancy are the same or higher and, possibly, how much higher than for women with a singleton pregnancy. 

According to old 1990 guidelines from the Institute of Medicine, women pregnant with twins should take the same dose of folic acid as women with a singleton pregnancy, which is 300 µg [15]. It needs to be noted, though, it is a lower dose than the one recommended in the recent years to prevent neural tube defects. According to Canadian guidelines, women pregnant with twins should take in total 1000 µg of folic acid (from food and supplements) and should not exceed this amount on their own [36]. It is worth mentioning that the 1000 µg dose of folic acid is a dose referred to as Tolerable Upper Intake Level (UL) for adults, including pregnant women [71]. The folic acid dose of 1000 µg per day for women with a twin pregnancy is also recommended by Goodnight et al. [37]. 

There are currently no data yet on the relationship between the folic acid intake/supplementation by women pregnant with twins and neural tube defects. However, statistical data indicate that the incidence of neural tube defects for twin pregnancies is higher than for singleton pregnancies and amounts to 2.3/1000 births [72]. In terms of folic acid supplementation, randomized controlled studies do not show any advantages of such a solution from the perspective of preventing pre-eclampsia (a frequent complication in twin pregnancies). In one of them, high doses of folic acid (4.0–5.1 mg daily) given to women pregnant with twins did not reduce the risk of this complication; in the treatment group, the incidence of pre-eclampsia was even higher than in the placebo group, although the differences found were not statistically significant [62]. In the other randomized study, no benefits were observed among women at a high risk of pre-eclampsia (including women pregnant with twins), who supplemented 4.0 mg folic acid/day [63]. Folic acid supplementation in pregnancy can also be associated with the newborns’ birth weight, although data in that respect usually apply to singleton pregnancies only [73]. The only study known to the author that related to women with a twin pregnancy in China demonstrated that, in women taking 400 µg folic acid before pregnancy and in the first trimester of pregnancy, the risk of a small-for-gestational-age infant was 55% lower and the risk of low birth weight was 50% lower, compared to women who did not supplement this vitamin [64]. 

#### 3.3.2. Vitamin D

It is believed that increased requirements and a higher risk of deficits in a twin pregnancy also applies to vitamin D [39,61], but due to insufficient studies on the actual requirements, the Institute of Medicine does not differentiate the recommended intake between singleton and twin pregnancies [74]. Although the scientific interest in vitamin D has been enormous in the recent years, it seems that, as opposed to other vitamins, the discussion here revolves more around its recommended blood concentration than its recommended intake. Considering the recommended serum 25(OH)D concentration proposed by some experts, which is at least 30 ng/mL, vitamin D deficiencies among pregnant women are common and apply to 99–100% women in Turkey [75], 69–95% in Central Europe [76,77], 52–85% in Southern Europe [78], 74% in the United States [79] and 63% in China [80].

Most studies indicate that vitamin D blood concentration in women with a multiple pregnancy is lower than in women with a singleton pregnancy. In Japanese women, throughout the entire pregnancy, the average 25(OH)D concentration in women pregnant with twins was considerably lower and, in the 36th week of pregnancy, amounted to 15.0 ng/mL as compared to 25.3 ng/mL in a singleton pregnancy [65]. In India, the average concentration in women pregnant with twins at birth was 5.7 ng/mL and, just like in the Japanese study, it was statistically significantly lower than in women with a singleton pregnancy (7.4 ng/mL). An analogical situation was observed for the infants’ cord blood. The average vitamin D concentration in twins and singleton infants amounted to 5.9 ng/mL and 9.1 ng/mL, respectively. At this point, it is worth emphasizing that despite large vitamin D deficits, there is currently no recommendations in India relating to dietary supplementation in pregnant women, regardless of whether this is a singleton or twin pregnancy [61]. A higher 25(OH)D blood concentration in women with a multiple pregnancy compared to women with a singleton pregnancy has so far been observed in an American study only (61 ng/mL vs. 39 ng/mL) [67]. In China, in spite of the fact that the vitamin D concentration in mothers pregnant with twins was quite high (on average 31.8 ng/mL), which could arise from the fact that all the women took vitamin–mineral supplements containing vitamin D, the cord blood concentrations were considerably lower than the mothers’ concentrations (on average 15.4 ng/mL) and as much as 78.5% of the newborns had vitamin D deficiency (concentration < 20 ng/mL) [66]. A study among American women with a twin pregnancy demonstrated that, although the mean 25(OH)D concentration was as high (33.1 ng/mL) as in the Chinese study, more than 40% of the women had less than 30 ng/mL of blood vitamin D. Compared to the vitamin D concentration below 30 ng/mL, concentration 30 ng/mL or above was associated with an 60% reduction in risk of preterm birth less than 35 weeks [60].

It is worth mentioning that the approach to vitamin D supplementation in pregnancy is very diverse on a global scale. According to the last consensus of world’s scientific organizations (2016), pregnant women are advised to supplement 600 IU/day [81], although WHO’s latest statement (2020) indicates that supplementation should only apply to women with suspected vitamin D deficiency and, in such a situation, the dose of 200 IU/day is sufficient [82]. The recommendations of the Polish Society of Gynecologists and Obstetricians subject the vitamin D dose to the patient’s BMI. For women with a normal body weight, 1500–2000 IU/day of vitamin D is recommended, while for obese women—after consultation with a physician—a higher dose of 4000 IU [83]. None of the above recommendations specifically refer to women with a multiple pregnancy, with only Canada and the USA expressing their opinion on the supplementation of this vitamin by women pregnant with twins. Based on the Canadian guidelines from Alberta Health Services, such women should intake 1200 IU of vitamin D and should not exceed 4000 IU per day [36], while according to the recommendations of the American Institute of Medicine, the dose of vitamin D should only amount to 200 IU [14]. Taking into account the currently common vitamin D deficiencies, the American guidelines seem to be questionable.

#### 3.3.3. Other Vitamins

In line with the majority of medical associations and WHO statements, routine use of multiple micronutrient supplements by all pregnant women is not recommended [27], but it needs to be remembered that this is a general recommendation not directed at multiple pregnancies. In low- and middle-income countries meta-analyses of randomized controlled trials and quasi-experimental studies showed improvement in several key birth outcomes in a singleton pregnancy, such as pre-term birth, small-for-gestational age and low birth weight with multiple micronutrient supplementation, compared to iron-folic supplementation [84]. Goodnight et al., claim that, in a twin pregnancy, it is advisable to take a micronutrient supplement [37], while other experts emphasize the need to supplement vitamin C at 150 mg per day due to its role in the synthesis of collagen, which is present in chorionic membranes, and thus it has a potential role in reducing the risk of a premature rupture of those membranes [15,37]. The Institute of Medicine recommends taking 50 mg of vitamin C per day after the 12th week of pregnancy [85]. When it comes to the remaining vitamins, there are currently no specific recommendations for their intake and dietary supplementation, but literature advises caution in terms of vitamin A, the requirement of which is probably not increased in a twin pregnancy. It is known that high doses of this vitamin can damage the fetal central nervous system and cause different congenital defects [17,36,39]. According to some experts, scientific data (based on studies in women with a singleton pregnancy) do not justify routine vitamin A supplementation, and the same applies to vitamins E, B1, B2 and K [86]. Without appropriate scientific studies, it is difficult to explicitly state that the same approach should be adopted for a twin pregnancy. 

The experts from Alberta Health Services in Canada suggest that, as part of perinatal care, pregnant women should receive help as to the choice of an appropriate supplement so that its daily dose contains 1000 µg folic acid, at least 27 mg of iron, 2.6 µg of vitamin B12 and at least 400 IU of vitamin D. At the same time, they do not recommend taking more than one vitamin–mineral supplement [36]. Luke, on the other hand, suggest that vitamin doses should not exceed 200% daily reference value [17].

### 3.4. Mineral Requirement

#### 3.4.1. Iron

Iron is an essential nutrient that is necessary for the normal course of pregnancy. It is estimated that, in a singleton pregnancy, the requirement for absorbed iron increases throughout the entire pregnancy by an average of 4.4 mg per day (0.8 mg per day at the beginning of pregnancy up to 7.5 mg in an advanced pregnancy) [87]. The European Food Safety Authority does not, however, recommend taking iron in excess of what is necessary for women who are not pregnant because in pregnancy, the absorption of this element is enhanced [88]. In a twin pregnancy, the iron requirement is 1.8 times higher compared to a singleton pregnancy. It stems from the additionally increased blood volume (by 10–20% compared to a singleton pregnancy), increased red blood count (up to the 20th week of pregnancy by 20–25% compared to a singleton pregnancy) and different needs of the mother and her children [15,50,68]. Anemia in women pregnant with twins occurs 2.4–4 times more frequently than in women with a singleton pregnancy and affects 30–45% of pregnant women in the third trimester [3,68,89]. Anemia in pregnancy is generally defined as a hemoglobin concentration below 11.0 g/dL in the first and third trimester of pregnancy and ≤10.5 g/dL in the second trimester [3], although according to some experts, the limit hemoglobin concentration in the third trimester is 10.5 g/dL [87,90]. Publications usually do not touch upon the issues of separate criteria for anemia in women pregnant with twins [91,92], but Shinar et al., suggest that in the second trimester, the cut-off point for hemoglobin concentration should be 9.7 g/dL because it is the best prognostic indicator for anemia [93].

It is commonly known that anemia in pregnancy increases the risk of pre-term birth and low birth weight [15,17,68,94,95]. It is estimated that, if anemia develops in women with a twin pregnancy at 12 weeks, the risk of pre-term birth increases to 29–68%, while if it happens at 16–18 weeks, the risk is three- to four-fold [96]. According to Luke, low hemoglobin concentrations lead to the adaptive enlargement of the placenta and can adversely affect the children’s susceptibility to hypertension in the subsequent years of their life [96]. A randomized study among 87 Israeli women pregnant with twins suffering from iron-deficiency anemia demonstrated that, in women who took 68 mg of elemental iron from the 16th week of pregnancy to 6 weeks postpartum, the hemoglobin and ferritin concentrations at 32 weeks and postpartum were statistically significantly higher than in a group taking 34 mg of iron. Therefore, the authors claim that women with anemia should preferably take a double dose of iron, although the same authors, in a different study carried out among women with a singleton pregnancy, did not demonstrate that such a dose significantly increases the hemoglobin and ferritin concentrations. At the same time, a study among women with a twin pregnancy did not show any difference in the duration of pregnancy and the newborns’ birth weight based on the iron dose [68]. The other randomized study was carried out in Egypt among 120 non-anemic women pregnant with twins in the first trimester who took 27 mg and 54 mg of elemental iron. It was found that both iron doses taken from the 12th to the 36th week of gestation maintained the normal hemoglobin and hematocrit concentrations, but the ferritin concentration in the double-dose group was higher [69]. The second study in Egypt also found no statistically significant difference in the hemoglobin concentrations between a single (27 mg) and a double dose (54 mg) of iron in non-anemic women with twin pregnancy [70]. In both studies, the higher doses, however, led to a higher incidence of the side effects (nausea/vomiting, or noncompliance, constipation, black staining of the stools) [69,70].

What are the iron intake/supplementation recommendations for women pregnant with twins, then, if we know from the latest studies that unnecessary iron supplementation may have adverse effects on the course of pregnancy, increasing the risk of pregnancy-induced hypertension, pre-eclampsia and gestational diabetes [39,97].

The latest position statement of the US Preventive Services Task Force is very cautious and indicates there are too little data to estimate the benefits and risks of routine iron supplementation in pregnant women in order to prevent the adverse course of pregnancy and experts from this organization do not take a different stand with regard to multiple pregnancies [98]. The same approach is adopted by the American College of Obstetricians and Gynecologists, and just like the previous opinion, does not refer to iron supplementation in women pregnant with twins [86,99]. Routine iron supplementation for all women is also not recommended in Great Britain. According to British experts, non-anemic women who are at a higher risk of iron deficiency, should be checked for the ferritin concentration already in early pregnancy and, if the concentration is lower than 30 μg/L, dietary supplementation needs to be started. Women with diagnosed iron-deficiency anemia should receive 100–200 mg of elemental iron per day [90]. In Poland, according to the latest recommendations of the Polish Society of Gynecologists and Obstetricians (2020) that take into account the scientific findings about the adverse impact of excessive iron intake, this element should only be taken by anemic women, while in non-anemic women with low ferritin, dietary supplementation with low iron doses can be recommended from the 16th week of pregnancy (up to 30 mg per day). None of the above guidelines refer specifically to women pregnant with twins [83].

As iron should not be used by pregnant women on their own, its presence in most vitamin-mineral supplements for pregnant women can be problematic. Based on the composition of prenatal supplements commercially available in Poland estimated by Wierzejska in 2019, 82% of them contained iron at doses of 14–60 mg, on average 28.5 mg [100]. At the same time, it is worth mentioning that, recently, the Panel on Dietary Supplements (an advisory body to the Chief Sanitary Inspector in Poland) has established the maximum iron dose in dietary supplements for pregnant women at 30 mg, so that the composition of some preparations will probably have to be changed. According to the Panel’s resolution, it is also recommended that such supplements should include the following warning: *“product for pregnant women, use after consulting a doctor”* [101]. However, Ru et al., pose a question in their publication whether the iron content in a standard prenatal supplement commercially available in the USA (27 mg) is sufficient for women with a twin pregnancy [3].

Only experts from North America raise the subject of iron supplementation in women pregnant with twins. The Institute of Medicine in the USA in 1990 considered that women with a multiple pregnancy should take 30 mg of elemental iron per day from the 12th week of gestation [85], while according to the Canadian guidelines, 30 mg of iron is the total daily requirement, of which 27 mg can be supplied by vitamin-mineral supplements and the rest with diet. Higher amounts of iron can only be prescribed by a physician [36]. In the face of the worldwide lack of specific guidelines for women pregnant with twins, a special approach to iron intake with diet and optimal supplementation is certainly required for women with a twin pregnancy who are on a plant-based diet, e.g., vegetarian or vegan.

#### 3.4.2. Calcium

Apart from iron, another mineral that is particularly important during pregnancy is calcium. However, available studies do not provide a conclusive answer to the question of whether calcium serum concentrations differ between women with a twin pregnancy and women with a singleton pregnancy. In an Indian study, it was demonstrated that the calcium blood concentration in women with a twin pregnancy at birth was statistically significantly lower than in women with a singleton pregnancy (8.7 mg/dL vs. 9.1 mg/dL), which resulted in the same lower calcium concentration in the newborns’ cord blood (9.6 mg/dL vs. 9.9 mg/dL) [61]. In a Japanese study, calcium concentrations determined in the 25th and 30th week of pregnancy in women pregnant with twins was higher than in women with a singleton pregnancy, but the difference blurred in the 36th week of pregnancy. At the same time, it was found that bone resorption markers in women pregnant with twins were statistically significantly higher compared to women with a singleton pregnancy [65], which also had been previously observed by Okah et al. [67]. Experts believe that this stems from a physiological mechanism that allows women pregnant with twins to meet the fetal calcium requirements and, at the same time, confirms that this element is extremely important in the maternal diet [65].

According to Canadian Alberta Health Services, a twin pregnancy requires additional amounts of calcium in the diet and the total calcium intake should amount to 2000–2500 mg per day [36]. The Institute of Medicine recommends supplementing 250 mg per day after the 12th week of gestation [85], while Luke argues that 3000 mg of calcium needs to be supplemented [52]. Such a high requirement for this nutrient would mean that the increased calcium absorption during pregnancy, which is caused by the higher concentration of the active metabolite of vitamin D (calcitriol), is not sufficient to cover the needs of both fetuses. At the same time, experts recommend that a single dose of a calcium preparation should not exceed 500 mg in order to ensure the maximum absorption of this element and minimize adverse reactions (flatulence, constipation). It is suggested that calcium should be taken in the form of single preparations and at least 2 h apart from taking a multi-nutrient preparation containing iron because these nutrients interact with each other, causing lower calcium bioavailability [36]. It is believed that calcium supplementation in a twin pregnancy is particularly important in very young women and in women who supply little calcium in their diet [17,37]. According to Luke, calcium supplementation has been proven to reduce the risk of gestational hypertension [96].

#### 3.4.3. Other Minerals

Some argue that, apart from a routine vitamin–mineral supplement, women pregnant with twins should be advised to take additional doses of magnesium and zinc because the requirements for these elements considerably rise [37], while others do not reckon these elements need supplementation [36]. A very important mineral component during pregnancy is, undoubtedly, iodine and most scientific societies recommend its supplementation at 150–200 µg per day [83,86,102]. It is unknown, however, whether such a dose is also appropriate for women pregnant with twins.

### 3.5. Other Dietary Recommendations

In the opinion of many experts, in women with a twin pregnancy, it is worth considering docosahexaenoic acid/eicosapentaenoic acid (DHA/EPA) supplementation at 300–500 mg per day [36,37]. It is generally believed that DHA supplementation reduces the risk of pre-term birth and low birth weight [17,83,103], which can be particularly important for a twin pregnancy. A meta-analysis of randomized studies demonstrated that omega-3 fatty acid supplementation by women with a singleton pregnancy reduced the risk of early pre-term delivery (<34 weeks of pregnancy) by 58%, any pre-term delivery by 17%, and significantly increased the birth weight of the newborn by 122 g [104]. In Poland, women at risk of a pre-term birth are advised to take 1000 mg DHA per day [83]. Notably, the European Committee allowed food producers to use the following health claim: *‘maternal DHA consumption contributes to normal brain and eye development in the fetus’,* which is indicative of the proven, beneficial role of this nutrient during pregnancy [105]. DHA accumulation in the central nervous system occurs after 20 weeks of gestation, so pre-term infants are at a particularly high risk for DHA deficiency [106,107]. Single studies indicate that the DHA concentration in the erythrocytes of women pregnant with twins is lower than in women with a singleton pregnancy, which leads to the assumption that the fetal DHA requirements might not be fully satisfied [108]. If, due to the low DHA intake in many countries of the world [109,110], supplementation is recommended for women with a singleton pregnancy [83,103], then it is even more reasonable when it comes to women with a multiple pregnancy, although the exact amount of this nutrient that would be optimal for them has yet to be established.

Out of all macronutrients, literature only points to the particular importance of protein because too little amino acids may adversely affect the development of the fetus and the placenta. It is proposed that 20% of energy in the diet of women pregnant with twins should come from proteins, 40% from carbohydrates and 40% from fats [14,15,17,37].

Many experts also emphasize the importance of dietary advice [23,36,40]. A study that estimated the frequency of such advice indicates that 63% of women pregnant with twins received such advice as part of standard care. According to 39% of them, the advice was very general and pointed to the need of having a well-balanced and healthy diet, while 40.5% women were advised to eat more protein. Less than 12% of the women said that the advice was concerned with eating fruit and vegetables, while women with diabetes were advised to consume less carbohydrates and sugars. As a consequence, women who followed the advice had a better diet [7]. Other studies demonstrated that women pregnant with twins who received dietary care more frequently gave birth to children with a normal birth weight. In one of such studies, women gained more weight, which reduced the risk of delivering an infant with low birth weight by 25% [14], while in the other study, intensive prenatal care resulted in a lower risk of delivering small-for-gestational-age infants by 40% [111]. Considering the prevalence of vegetarianism among young people [112], women pregnant with twins who are on a plant-based diet would need special dietary care due to the increased risk of deficiency of some nutrients.

## 4. Discussion

Studies among women with a singleton pregnancy show that a substantial number of women do not meet the guidelines as to the intake of some vitamins and minerals [101,113]. Some claim that no single micronutrient is responsible for the adverse pregnancy outcomes and supplementing or correcting one deficiency will not be very effective as long as other deficiencies exist [86].

Insufficient data on the nutritional needs of women pregnant with twins were already emphasized in literature 20 years ago and almost nothing has changed since that time. Therefore, it is impossible to determine whether deficiencies in their diets are more frequent and more intensified than in the diets of women with a singleton pregnancy. At the same time, without studies relating to the body saturation with nutrients during pregnancy, also considering the pre-pregnancy nourishment of women, it is difficult to estimate the actual body requirements and reliably state whether the approach to potential supplementation should differ between women with a singleton pregnancy and women with a multiple pregnancy. Furthermore, due to differences in access to food between high-income and low-income countries and the policy of individual countries in terms of food fortification with vitamins and minerals, the risk of nutrient deficiencies on a global scale is varied.

At the moment, experts are in agreement that women pregnant with twins should increase their energy intake. However, such a universal recommendation also requires further discussion because it does not include the pre-pregnancy diet and BMI of women. It is not uncommon that it turns out to be necessary to maintain the diet’s energy value or even reduce it. An excessive body weight gain during pregnancy, as it is frequently observed, even more so justifies the need of an individual approach to energy requirements. Perhaps, the time is coming for a serious verification of the existing established standards of energy intake for pregnant women in developed countries due to the changing lifestyle and good nourishment of most women at reproductive age. As a meta-analysis of studies on energy intake and body weight gain carried out by Jebeile et al., indicates, the energy intake during pregnancy in women with a singleton pregnancy rises by only 113 kcal daily, with the total energy intake often below 2000 kcal. Despite that, only few studies reported too low a body weight gain [42]. Wider discussion would also be required with regard to the theoretical need to accumulate energy in the form of fatty tissue by women with normal or excessive pre-pregnancy body weight and, to the same extent, these doubts apply to women pregnant with twins.

Furthermore, it needs to be acknowledged that even the most precisely established energy and nutrient requirements do not guarantee that, in practice, this will be highly useful for the mothers and will contribute to better pregnancy outcomes. This would require that women have broad knowledge on nutrition and the energy value of meals. When it comes to single food products, this issue seems to be easier because unit packages of food contain data on the nutritional value of 100 g of the product. For meals and complex dishes, estimating their energy value is difficult and, without consulting an experienced dietician, balancing the diet in pregnancy seems to be unrealistic. Besides, the sole information about the necessity to increase the energy intake without additional explanations on how to put it into everyday practice can be confusing for women and lead to increased consumption of products without a high nutritional value. Given that a twin pregnancy is associated with a high risk of low birth weight, it is advisable that perinatal care should include educational meetings carried out by professional who can teach patients what they should eat to supply the optimum amount of calories and nutrients.

## 5. Conclusions

Rarely are twin pregnancies a field of study in nutritional science. In the light of the increasing number of such pregnancies and the risk of complications, which can also be to some extent associated with nutrition, scientific studies are needed that would examine the maternal saturation with nutrients. Such studies will help to establish to what extent the requirements for women pregnant with twins are different than those for women with a singleton pregnancy and will allow for developing supplementation guidelines.

Coming back to the question in the title of the article, it should be stated that the issue of nutritional needs of women pregnant with twins has been somewhat neglected by nutrition science.

## Figures and Tables

**Table 1:** Characteristics of studies on the concentration of nutrients and their supplementation in women pregnant with twins.

Authors	Study Design	Participants	Type of Nutrients	Duration of the Study	Results
Corsi et al., 2020 [62]	Randomized Controlled Trial	428 women with twin pregnancy	Folic acid (4.0–5.1 mg vs. placebo)	II–III trimester of pregnancy	No effect of a high dose of folic acid in the prevention of pre-eclampsia
Wen et al., 2018 [63]	Randomized Controlled Trial	2464 women with singleton or twin pregnancies	Folic acid (4.0 mg vs. placebo)	From 8 weeks of pregnancy to delivery	No effect of a high dose of folic acid in the prevention of pre-eclampsia
Zhang et al., 2020 [64]	Cross-sectional study	28,174 women with singleton or twin pregnancies	Folic acid (0.4 mg)	From 12 weeks before pregnancy to the end of the first trimester of pregnancy	15–55% reduction in small-for-gestational-age infant. 18–50% reduction in low birth weight infant. Increased birth weight by 17.3–166.3 g
Nakayama et al., 2011 [65]	Cross-sectional study	322 women with singleton or twin pregnancies	Vitamin D, calcium	From 10 to 36 weeks of pregnancy	Concentration of 1,25(OH)_2_D and 25(OH)D in women with twin pregnancy were lower than in women with singleton pregnancy. Serum calcium concentration in women with twin in the 25th and 30th week of pregnancy were higher than in women with singleton pregnancy. Concentration of bone resorption markers in women with twin pregnancy were higher than in women with singleton pregnancy
Goswami et al., 2016 [61]	Cross-sectional study	100 women with singleton or twin pregnancies	Vitamin D, calcium	Time of childbirth	Concentration of 25(OH)D and calcium in women with twin pregnancy were lower than in women with singleton pregnancy
Li et al., 2021 [66]	Prospective subcohort study	72 women with twin pregnancy	Vitamin D	III trimester of pregnancy	Twin neonates were at high risk of vitamin D deficiency
Bodnar et al., 2013 [60]	Cohort study	661 women with twin pregnancy	Vitamin D	From 24 to 28 weeks of pregnancy	60% reduction in preterm birth (< 35 weeks of pregnancy) at a concentration of 25(OH)D ≥ 30 ng/mL compared to a concentration < 30 ng/mL
Okah et al., 1996 [67]	Cross-sectional study	47 women with singleton or twin pregnancies	Vitamin D	III trimester of pregnancy	Concentration of 25(OH)D in women with twin pregnancy were higher than in women with singleton pregnancy. Concentration of bone resorption markers in women with twin pregnancy were higher than in women with singleton pregnancy
Shinar et al., 2017 [68]	Randomized Controlled Trial	172 iron-deficient women with twin pregnancy	Iron (34 mg of ferrous sulfate vs. 68 mg)	From 16 weeks of pregnancy to 6 weeks postpartum	The dose of 68 mg of elemental ferrous sulfate is beneficial for iron-deficient women with twin pregnancies
Ali et al., 2017 [69]	Randomized Controlled Trial	120 non-anemic women with twin pregnancy	Iron (27 mg elemental iron vs. 54 mg)	From 12 to 36 weeks of pregnancy	The effectiveness of the dose of 54 mg elemental iron and 27 mg is compared in the prevention of anemia
Abbas et.al., 2020 [70]	Randomized Controlled Trial	450 non-anemic women with twin pregnancy	Iron (27 mg elemental iron vs. 54 mg)	From 12 weeks of pregnancy to delivery	Compared to the single dose, the double supplemental iron dose has not significantly lowered the incidence of iron deficiency anemia, nor has contributed to increase of the hemoglobin concentration in pregnancies not complicated by iron deficiency anemia.

## Data Availability

Not applicable.

## References

[1] Martin J.A., Hamilton B.E., Osterman M.J., Curtin S.C., Mathews T.J. (2015). Final data for 2013. Natl. Vital Stat. Rep..

[2] Muggli E., Halliday J.L. (2007). Folic acid and risk of twinning: A systematic review of the recent literature, July 1994 to July 2006. Med. J. Aust..

[3] Ru Y., Pressman E.K., Cooper E.M., Guillet R., Katzman P.J., Kent T.R., Bacak S.J., O’Brien K.O. (2016). Iron deficiency and anemia are prevalent in women with multiple gestations. Am. J. Clin. Nutr..

[4] Bricker L., Reed K., Wood L., Neilson J.P. (2015). Nutritional advice for improving outcomes in multiple pregnancies (Review). Cochrane Database Syst. Rev..

[5] Smits J., Monden C. (2011). Twinning across the developing world. PLoS ONE.

[6] Lal A.K., Kominiarek M.A. (2015). Weight gain in twin gestations: Are the Institute of Medicine guidelines optimal for neonatal outcomes?. J. Perinatol..

[7] Whitaker K.M., Baruth M., Schlaff R.A., Talbot H., Connolly C.P., Liu J., Wilcox S. (2019). Provider advice on physical activity and nutrition in twin pregnancies: A cross-sectional electronic. BMC Pregnancy Childbirth.

[8] Committee on Practice Bulletins—Obstetrics, Society for Maternal–Fetal Medicine (2016). Practice Bulletin No. 169: Multifetal Gestations: Twin, Triplet, and Higher-Order Multifetal Pregnancies. Obstet. Gynecol..

[9] Esteves-Pereira A.P., da Cunha A.J.L.A., Nakamura-Pereira M., Moreira M.E., Domingues R.M., Viellas E.F., Leal M., da Gama S.G. (2021). Twin pregnancy and perinatal outcomes: Data from ‘Birth in Brazil Study. PLoS ONE.

[10] Statystyczne R. (1991). Demografia 1991. Główny Urząd Statystyczny.

[11] (2019). The Demographic Yearbook of Poland 2019.

[12] Santana D.S., Surita F.G., Cecatt J.G. (2018). Multiple pregnancy: Epidemiology and association with maternal and perinatal morbidity. Rev. Bras. Ginecol. Obstet..

[13] Grantz K.L., Kawakita T., Lu Y.L., Newman R., Berghella V., Caughey A., SMFM Research Committee (2019). SMFM Special Statement: State of the science on multifetal gestations: Unique considerations and importance. Am. J. Obstet. Gynecol..

[14] Brown J.E., Carlson M. (2000). Nutrition and multifetal pregnancy. J. Am. Diet. Assoc..

[15] Roselló-Soberon M.E., Fuentes-Chaparro L., Casanueva E. (2005). Twin pregnancies: Eating for three? Maternal nutrition update. Nutr. Rev..

[16] Ramiro-Cortijo D., de la Calle M., Rodríguez-Rodríguez P., López de Pablo A.L., López-Giménez M.R., Aguilera Y., Martín-Cabrejas M.A., del Carmen González M., Arribas S.M. (2020). Maternal antioxidant status in early pregnancy and development of fetal complications in twin pregnancies: A pilot study. Antioxidants.

[17] Luke B. (2005). Nutrition and multiple gestation. Semin. Perinatol..

[18] Nazer J., Aguila A., Cifuentes L.R. (2006). The frequency of twin pregnancies increased in a Chilean hospital associated with periconceptional flour folic acid supplementation. Rev. Méd. Chile.

[19] Lumley J., Watson L., Watson M., Bower C. (2001). Periconceptional supplementation with folate and/or multivitamins for preventing neural tube defects. Cochrane Database Syst. Rev..

[20] Moussa H.N., Nasab S.H., Haidar Z.A., Blackwell S.C., Sibai B.M. (2016). Folic acid supplementation: What is new? Fetal, obstetric, long-term benefits and risks. Future Sci..

[21] Hutcheon J.A., Platt R.W., Abrams B., Braxter B.J., Eckhardt C.L., Himes K.P., Bodnar L.M. (2018). Pregnancy weight gain by gestational age in women with uncomplicated dichorionic twin pregnancies. Paediatr. Perinat. Epidemiol..

[22] Bodnar L.M., Himes K.P., Abrams B., Lash T.L., Parisi S.M., Eckhardt C.L., Braxter B.J., Minion S., Hutcheon J.A. (2019). Gestational weight gain and adverse birth outcomes in twin pregnancies. Obstet. Gynecol..

[23] Pettit K.E., Lacoursiere D.Y., Schrimmer D.B., Alblewi H., Moore T.R., Ramos G.A. (2015). The association of inadequate mid-pregnancy weight gain and preterm birth in twin pregnancies. J. Perinatol..

[24] Narang K., Szymanski L.M. (2020). Multiple gestations and hypertensive disorders of pregnancy: What do we know?. Curr. Hypertens. Rep..

[25] Jarosz M., Rychlik E., Stoś K., Charzewska J. (2020). Nutrition Standards for the Population of Poland and Their Application.

[26] Skrypnik D., Moszak M., Wender-Ozegowska E., Bogdanski P. (2021). Comparison of Polish and international guidelines on diet supplements in pregnancy—Review. Ginekol. Pol..

[27] World Health Organization (2020). WHO Antenatal Care Recommendations for a Positive Pregnancy Experience: Nutritional Interventions Update: Multiple Micronutrient Supplements during Pregnancy.

[28] Most M., Dervis S., Haman F., Adamo K.B., Redman L.M. (2019). Energy intake requirements in pregnancy. Nutrients.

[29] Agostoni C., Bresson J.L., Fairweather-Tait S. (2013). EFSA Panel on Dietetic Products, Nutrition and Allergies (NDA): Scientific opinion on dietary reference values for energy. EFSA J..

[30] (2004). Food and Agriculture Organization of the United Nations/World Health Organization/United Nations University (FAO/WHO/UNU). Human Energy Requirements, Report of a Joint FAO/WHO/UNU Expert Consultation, Rome.

[31] Butte N.F., King J.C. (2005). Energy requirements during pregnancy and lactation. Public Health Nutr..

[32] Shinagawa S., Suzuki S., Chihara H., Otsubo Y., Takeshita T., Araki T. (2005). Maternal basal metabolic rate in twin pregnancy. Gynecol. Obstet. Investig..

[33] Gandhi M., Gandhi R., Mack L.M., Shypailo R., Adolph A.L., Puyau M.R., Wong W.W., Deter R.L., Sangi-Haghpeykar H., Lee W. (2018). Estimated energy requirements increase across pregnancy in healthy women with dichorionic twins. Am. J. Clin. Nutr..

[34] Blumfield M.L., Hure A.J., Macdonald-Wicks L., Smith R., Collins C.E. (2012). Systematic review and meta-analysis of energy and macronutrient intakes during pregnancy in developed countries. Nutr. Rev..

[35] Dubois S., Doughtery C., Duquette M.P., Hanley M.P.D., Moutquin J.M. (1991). Twin pregnancy: The impact of the Higgins Nutrition Intervention Program on maternal and neonatal outcomes. Am. J. Clin. Nutr..

[36] Alberta Health Services (2018). Nutrition Guideline Pregnancy: Multiples. https://www.albertahealthservices.ca/assets/info/nutrition/if-nfs-ng-pregnancy-multiples.pdf.

[37] Goodnight W., Newman R. (2009). Optimal nutrition for improved twin pregnancy outcome. Obstet. Gynecol..

[38] Most J., Amant M.S., Hsia D., Altazan A.D., Thomas D.M., Gilmore L.A., Vallo P.M., Beyl R.A., Ravussin E., Redman L.M. (2019). Evidence-based recommendations for energy intake in pregnant women with obesity. J. Clin. Investig..

[39] Zgliczynska M., Kosinska-Kaczynska K. (2021). Micronutrients in multiple pregnancies-the knowns and unknowns: A systematic review. Nutrients.

[40] Roem K. (2003). Nutritional management of multiple pregnancies. Twin Res..

[41] Morley R., Umstad M.P., Bond J., Moore V.M., Owens J.A., Dwyer T., Carlin J.B. (2006). Maternal dietary intake in twin pregnancies: Does it diminish towards term?. Twin Res. Hum. Genet..

[42] Jebeile H., Mijatovic J., Louie J.C.Y., Prvan T., Brand-Miller J.C. (2016). Systematic review and meta-analysis of energy intake and weight gain in pregnancy. Am. J. Obstet. Gynecol..

[43] Koletzko B., Bauer C.P., Bung P., Cremer M., Flothkötter M., Hellmers C., Kersting M., Krawinkel M., Przyrembel H., Rasenack R. (2013). German national consensus recommendations on nutrition and lifestyle in pregnancy by the ‘Healthy Start—Young Family Network’. Ann. Nutr. Metab..

[44] Diemert A., Lezius S., Pagenkemper M., Hansen G., Drozdowska A., Hecher K., Arck P., Zyriax B.C. (2016). Maternal nutrition, inadequate gestational weight gain and birth weight: Results from a prospective birth cohort. BMC Pregnancy Childbirth.

[45] Gandhi M. (2020). Why Pregnancy weight gain guidelines need to differ for multiple versus single pregnancies. Curr. Nutr. Rep..

[46] Obata S., Shimura M., Misumi T., Nakanishi S., Shindo R., Miyagi E., Aoki S. (2021). Weight gain during twin pregnancy with favorable pregnancy outcomes in Japan: A retrospective investigation for new criteria based on perinatal registry data. PLoS ONE.

[47] Pécheux O., Garabedian C., Drumez E., Mizrahi S., Cordiez S., Deltombe S., Deruelle P. (2019). Maternal and neonatal outcomes according to gestational weight gain in twin pregnancies: Are the Institute of Medicine guidelines associated with better outcomes?. Eur. J. Obstet. Gynecol. Reprod. Biol..

[48] Goldstein R.F., Abell S.K., Ranasinha S., Misso M.L., Boyle J.A., Harrison C.L., Black M.H., Li N., Hu G., Corrado F. (2018). Gestational weight gain across continents and ethnicity: Systematic review and meta-analysis of maternal and infant outcomes in more than one million women. BMC Med..

[49] Bodnar L.M., Pugh S.J., Abrams B., Himes K.P., Hutcheon J.A. (2014). Gestational weight gain in twin pregnancies and maternal and child health: A systematic review. J. Perinatol..

[50] Hinkle S.N., Hediger M.L., Kim S., Albert P.S., Grobman W., Newman R.B., Wing D.A., Grewal J., Zhang C., Louis G.M.B. (2017). Maternal weight gain and associations with longitudinal fetal growth in dichorionic twin pregnancies: A prospective cohort study. Am. J. Clin. Nutr..

[51] Luke B., Leurgans S. (1996). Maternal weight gains in ideal twin outcomes. J. Am. Diet. Assoc..

[52] Luke B. (2015). Nutrition for multiples. Clin. Obstet. Gynecol..

[53] Institute of Medicine and National Research Council (2009). Weight Gain during Pregnancy: Reexamining the Guidelines.

[54] Wierzejska R., Wojda B. (2019). Pre-pregnancy nutritional status versus maternal weight gain and neonatal size. Roczn. Państw. Zakl. Hig..

[55] Fox N.S., Saltzman D.H., Kurtz H., Rebarber A. (2011). Excessive weight gain in term twin pregnancies: Examining the 2009 Institute of Medicine definitions. Obstet. Gynecol..

[56] Ozcan T., Bacak S.J., Zozzaro-Smith P., Li D., Sagcan S., Seligman N., Glantz C.J. (2017). Assessing weight gain by the 2009 Institute of Medicine guidelines and perinatal outcomes in twin pregnancy. Matern. Child Health J..

[57] Scott C., Andersen C.T., Valdez N., Mardones F., Nohr E.A., Poston L., Loetscher K.C., Abrams B. (2014). No global consensus: A cross-sectional survey of maternal weight policies. BMC Pregnancy Childbirth.

[58] National Institute for Health and Clinical Excellence (2010). Weight management before, during, and after pregnancy. NICE Public Health Guid..

[59] Lutsiv O., Hulman A., Woolcott C., Beyene J., Giglia L., Armson A., Dodds L., Neupane B., McDonald S.D. (2017). Examining the provisional guidelines for weight gain in twin pregnancies: A retrospective cohort study. BMC Pregnancy Childbirth.

[60] Bodnar L.M., Rouse D.J., Momirova V., Peaceman A.M., Sciscione A., Spong C.Y., Varner M.W., Malone F.D., Iams J.D., Mercer B.M. (2013). Maternal 25-hydroxyvitamin D and preterm birth in twin gestations. Obstet. Gynecol..

[61] Goswami D., Rani R., Saxena A., Arora M.S., Batra S., Sreenivas V. (2016). Maternal and neonatal vitamin-D status in twin versus singleton pregnancies. J. Obstet. Gynaecol. Res..

[62] Corsi D.J., Gaudet L.M., El-Chaar D., White R.R., Rybak N., Harvey A., Muldoon K., Wen S.W., Walker M. (2022). Effect of high-dose folic acid supplementation on the prevention of preeclampsia in twin pregnancy. J. Matern. Fetal Neonatal Med..

[63] Wen S.W., White R.R., Rybak N., Gaudet L.M., Robson S., Hague W., Simms-Stewart D., Carroli G., Smith G., Fraser W.D. (2018). Effect of high dose folic acid supplementation in pregnancy on pre-eclampsia (FACT): Double blind, phase III, randomised controlled, international, multicentre trial. BMJ.

[64] Zhang B., Shang S., Li S., Mi B., Li M., Shi G., Ma M., Wang Q., Yan H., Dang S. (2020). Maternal folic acid supplementation and more prominent birth weight gain in twin birth compared with singleton birth: A cross-sectional study in northwest China. Public Health Nutr..

[65] Nakayama S., Yasui T., Suto M., Sato M., Kaji T., Uemura H., Maeda K., Irahara M. (2011). Differences in bone metabolism between singleton pregnancy and twin pregnancy. Bone.

[66] Li X., Jiaxiao Y., Wen L., Li Q., Yan J., Tian J., Tong C., Tong Q., Qi H., Saffery R. (2021). Vitamin D status in women with dichorionic twin pregnancies and their neonates: A pilot study in China. BMC Pregnancy Childbirth.

[67] Okah F.A., Tsang R.C., Sierra R., Brady K.K., Specker B.L. (1996). Bone turnover and mineral metabolism in the last trimester of pregnancy: Effect of multiple gestation. Comp. Study Obstet. Gynecol..

[68] Shinar S., Skornick-Rapaport A., Maslovitz S. (2017). Iron supplementation in twin pregnancy—The benefit of doubling the iron dose in iron deficient pregnant women: A randomized controlled trial. Twin Res. Hum. Genet..

[69] Ali M.K., Abbas A.M., Abdelmagied A.M., Mohammed G.E., Abdalmageed O.S. (2017). A randomized clinical trial of the efficacy of single versus double-daily dose of oral iron for prevention of iron deficiency anemia in women with twin gestations. J. Matern. Fetal Neonatal Med..

[70] Abbas A.M., Elhalwagy M.M., Afifi K., Ibrahim K., Sweed M.S. (2020). Single vs. double dose iron supplementation for prevention of iron deficiency anemia in twin pregnancy: A randomized controlled clinical trial. Open J. Obstet. Gynecol..

[71] European Food Safety Authority (2014). Scientific opinion on dietary reference values for folate. EFSA J..

[72] Ross V., Reidy K., Doyle L.W., Palma-Dias R., Umstad M.P. (2018). Outcome of twin pregnancies complicated by a neural tube defect. Twin Res. Hum. Genet..

[73] Hodgetts V.A., Morris R.K., Francis A., Gardosi J., Ismail K.M. (2015). Effectiveness of folic acid supplementation in pregnancy on reducing the risk of small-for-gestational age neonates: A population study, systematic review and meta-analysis. BJOG Int. J. Obstet. Gynaecol..

[74] Ross A.C., Taylor C.L., Yaktine A.L., Del Valle H.B., Institute of Medicine (US) (2010). Committee to Review Dietary Reference Intakes for Calcium and Vitamin D.

[75] Halicioglu O., Aksit S., Koc F., Sezin A., Akman S.A., Albudak E., Yaprak I., Coker I., Colak A., Ozturk C. (2012). Vitamin D deficiency in pregnant woman and their neonates in spring time in western Turkey. Paediatr. Perinat. Epidemiol..

[76] Gellert S., Ströhle A., Bitterlich N., Hahn A. (2017). Higher prevalence of vitamin D deficiency in German pregnant women compared to non-pregnant women. Arch. Gynecol. Obstet..

[77] Wierzejska R., Jarosz M., Bachanek M., Sawicki W. (2020). Gestational vitamin D concentration and other risk factors versus fetal femur length. J. Matern. Fetal Neonatal Med..

[78] Rodriguez A., García-Esteban R., Basterretxea M., Lertxundi A., Rodríguez-Bernal C., Iñiguez C., Rodriguez-Dehli C., Tardón A., Espada M., Sunyer J.E. (2015). Associations of maternal circulating 25-hydroxyvitamin D3 concentration with pregnancy and birth outcomes. BJOG.

[79] Nobles C.J., Markenson G., Chasan-Taber L. (2015). Early pregnancy vitamin D status and risk for adverse maternal and infant outcomes in a bi-ethnic cohort: The Behaviors Affecting Baby and You (B.A.B.Y.) study. Br. J. Nutr..

[80] Wang C., Gao J.S., Yu S.L., Qiu L., Zeng L., Wang D.H. (2016). Correlation between neonatal vitamin D level and maternal vitamin D level. Zhongguo Dang Dai Er Ke Za Zhi.

[81] Craig F.M., Nick S., Kiely M., Specker B.L., Thacher T.D., Ozono K., Michigami T., Tiosano D., Mughal M.Z., Mäkitie O. (2016). Global consensus recommendations on prevention and management of nutritional rickets. J. Clin. Endocrinol. Metab..

[82] World Health Organization (2020). WHO Antenatal Care Recommendations for a Positive Pregnancy Experience: Nutritional Interventions Update: Vitamin D Supplements during Pregnancy.

[83] Zimmer M., Sieroszewski P., Oszukowski P., Huras H., Fuchs T., Pawłosek A. (2020). Rekomendacje Polskiego Towarzystwa Ginekologów i Położników dotyczące suplementacji u kobiet ciężarnych. Ginekol. I Perinatol. Prakt..

[84] Oh C., Keats E.C., Bhutta Z.A. (2020). Vitamin and mineral supplementation during pregnancy on maternal, birth, child health and development outcomes in low- and middle-income countries: A systematic review and meta-analysis. Nutrients.

[85] National Academy of Sciences (1990). Nutrition during Pregnancy.

[86] Santander Ballestín S., Giménez Campos M.I., Ballestín Ballestín J., Luesma Bartolomé M.J. (2021). Is supplementation with micronutrients still necessary during pregnancy? A review. Nutrients.

[87] Means R.T. (2020). Iron deficiency and iron deficiency anemia: Implications and impact in pregnancy, fetal development, and early childhood parameters. Nutrients.

[88] EFSA Panel on Dietetic Products, Nutrition and Allergies (NDA) (2015). Scientific Opinion on Dietary Reference Values for iron. EFSA J..

[89] Tekgül N., Yamazhan M. (2019). The effects of maternal anemia in pregnant women with respect to the newborn weight and the placental weight in the delivery room. J. Pediatr. Res..

[90] Pavord S., Myers B., Robinson S., Allard S., Strongand J., Oppenheimer C. (2012). UK guidelines on the management of iron deficiency in Pregnancy. Br. J. Haematol..

[91] Daru J., Allotey J., Peña-Rosas J.P., Khan K.S. (2017). Serum ferritin thresholds for the diagnosis of iron deficiency in pregnancy: A systematic review. Transfus. Med..

[92] Milman N., Taylor C.L., Merkel J., Brannon P.M. (2017). Iron status in pregnant women and women of reproductive age in Europe. Am. J. Clin. Nutr..

[93] Shinar S., Shapira U., Maslovitz S. (2018). Redefining normal hemoglobin and anemia in singleton and twin pregnancies. Int. J. Gynaecol. Obstet..

[94] Ru Y., Pressman E.K., Guillet R., Katzman P.J., Bacak S.J., O’Brien K.O. (2018). Predictors of anemia and iron status at birth in neonates born to women carrying multiple fetuses. Pediatr. Res..

[95] Zulfiqar H., Shah I.U., Sheas M.N., Ahmed Z., Ejaz U., Ullah I., Saleem S., Imran M., Hameed M., Akbar B. (2021). Dietary association of iron deficiency anemia and related pregnancy outcomes. Food Sci. Nutr..

[96] Luke B. (2005). Nutrition in multiple gestations. Clin. Perinatol..

[97] Milman N. (2011). Iron in pregnancy: How do we secure an appropriate iron status in the mother and child?. Ann. Nutr. Metab..

[98] Siu A.L., on behalf of the U.S. Preventive Services Task Force (2015). Screening for iron deficiency anemia and iron supplementation in pregnant women to improve maternal health and birth outcomes: U.S. Preventive Services Task Force Recommendation Statement. Ann. Intern. Med..

[99] American College of Obstetricians and Gynecologists (2008). Practice bulletin number 95—Anemia in pregnancy. Obstet. Gynecol..

[100] Wierzejska R. (2021). Evaluation of prenatal vitamin-mineral preparations in the context of recommended dietary supplementation. Are pregnant women supplied with what they should get?. Rocz. Panstw. Zakl. Hig..

[101] Uchwała nr 20/2019 Zespołu do spraw Suplementów Diety z 13 Grudnia 2019 w Sprawie Wyrażenia Opinii Dotyczącej Maksymalnej Dawki Żelaza w Zalecanej Dziennej Porcji w Suplementach Diety. www.gov.pl/web/gis/zespol-do-spraw-suplementow-diety.

[102] Jun S., Gahche J.J., Potischman N., Dwyer J.T., Guenther P.M., Sauder K.A., Bailey R.L. (2020). Dietary supplement use and its micronutrient contribution during pregnancy and lactation in the United States. Obstet. Gynecol..

[103] Dębski R., Karowicz-Bilińska A., Oszukowski P., Paszkowski T., Spaczyński M. (2014). Rekomendacje Polskiego Towarzystwa Ginekologicznego dotyczące zastosowania suplementacji kwasem dokozaheksaenowym w profilaktyce porodu przedwczesnego. Ginekol. Pol..

[104] Kar S., Wong M., Rogozinska E., Thangaratinam S. (2016). Effects of omega-3 fatty acids in prevention of early preterm delivery: A systematic review and meta-analysis of randomized studies. Eur. J. Obstet. Gynecol. Reprod. Biol..

[105] (2011). Commission Regulation (EU) No 440/2011 of 6 May 2011 on the authorisation and refusal of authorisation of certain health claims made on foods and referring to children’s development and health. Off. J. Eur. Union.

[106] Brouwer-Brolsma E.M., van de Rest O., Godschalk R., Zeegers M.P.A., Gielen M., de Groot R.H.M. (2017). Associations between maternal long-chain polyunsaturated fatty acid concentrations and child cognition at 7 years of age: The MEFAB birth cohort. Prostaglandins Leukot. Essent. Fat. Acids.

[107] Gellert S., Schuchardt J.P., Hahn A. (2016). Higher omega-3 index and DHA status in pregnant women compared to lactating women—Results from a German nation-wide cross-sectional study. Prostaglandins Leukot. Essent. Fat. Acids.

[108] McFadyen M., Farquharson J., Cockburn F. (2003). Maternal and umbilical cord erythrocyte omega-3 and omega-6 fatty acids and haemorheology in singleton and twin pregnancies. Arch. Dis. Child Fetal Neonatal. Ed..

[109] von Schacky C. (2020). Omega-3 fatty acids in pregnancy—The case for a target omega-3 index. Nutrients.

[110] Wierzejska R., Jarosz M., Wojda B., Siuba-Strzelińska M. (2018). Dietary intake of DHA during pregnancy: A significant gap between the actual intake and current nutritional recommendations. Rocz. Panstw. Zakl. Hig..

[111] Zhang B.Y., Li M.M., Liu A.M., Wu W.T., Guo H.Y., Gao X.Y., Wu C.L., Shang S.H., Yan H., Dang S.N. (2020). The association between the frequency of prenatal care in childbearing aged women and risk of small for gestational age among neonatal twins in Shaanxi Province. Zhonghua Yu Fang Yi Xue Za Zhi.

[112] Sebastiani G., Barbero A.H., Borrás-Novell C., Casanova M.A., Aldecoa-Bilbao V., Andreu-Fernández V., Tutusaus M.P., Martínez S.F., Roig M.D.G., García-Algar O. (2019). The effects of vegetarian and vegan diet during pregnancy on the health of mothers and offspring. Nutrients.

[113] Jankowska A., Grzesiak M., Krekora M., Dominowska J., Jerzynska J., Kałuzny P., Wesołowska E., Szadkowska-Stanczyk I., Trafalska E., Kaleta D. (2021). Determinants of the essential elements and vitamins intake and status during pregnancy: A descriptive study in Polish mother and child cohort. Nutrients.

